# Factors associated with mortality in patients undergoing coronary artery bypass grafting**^1^**

**DOI:** 10.1590/1518-8345.0708.2748

**Published:** 2016-08-08

**Authors:** Cintia Koerich, Gabriela Marcellino de Melo Lanzoni, Alacoque Lorenzini Erdmann

**Affiliations:** 2 Doctoral Student, Centro de Ciências da Saúde, Universidade Federal de Santa Catarina, Florianópolis, SC, Brazil. RN, Hospital Infantil Joana de Gusmão, Secretaria do Estado da Saúde de Santa Catarina, São José, SC, Brazil.; 3 PhD, Adjunct Professor, Centro de Ciências da Saúde, Universidade Federal de Santa Catarina, Florianópolis, SC, Brazil.; 4 PhD, Full Professor, Centro de Ciências da Saúde, Universidade Federal de Santa Catarina, Florianópolis, SC, Brazil.

**Keywords:** Mortality, Health Management, Thoracic Surgery, Hospitals

## Abstract

**Objective:**

to investigate the factors associated with mortality in patients undergoing coronary artery bypass grafting in a cardiovascular referral hospital in Santa Catarina.

**Method:**

quantitative, exploratory, descriptive and retrospective study. The medical records of 1447 patients, from 2005 to 2013, were analyzed for statistically related variables, these being: profile, hospitalization diagnosis, risk factors for coronary artery disease, complications recorded during the hospitalization, length of hospitalization and cause of death.

**Results:**

the mortality rate was 5.3% during the study period. Death was more common in females and those of black skin color, with a mean age of 65 years. Acute myocardial infarction was the most common hospitalization diagnosis. The majority of the complications recorded during hospitalization were characterized by changes in the cardiovascular system, with longer hospitalization periods being directly related to death from septic shock.

**Conclusion:**

the data provide subsidies for nursing work with preventive measures and early detection of complications associated with coronary artery bypass grafting. This reinforces the importance of using the data as quality indicators, aiming to guarantee care guided by reliable information to guide managers in planning patient care and high complexity health services.

## Introduction

Coronary artery bypass grafting (CABG) is considered one of the most frequent cardiac surgeries performed in the Brazilian National Health System (SUS) representing 77% of all surgeries performed in both public hospitals and philanthropic or private hospitals^1^. The decision for surgery is based on an individual analysis, taking into account factors such as the degree of angina, ventricular function, ischemic burden and coronary anatomy. Individuals with multivessel coronary disease and ventricular dysfunction, left main coronary artery obstruction or large areas of ischemia usually receive an indication for CABG^2^. 

Over the years CABG has received two essential contributions for the improvement of surgical outcomes throughout the world, both of which were introduced by Brazilian surgeons, namely: the technique of myocardial revascularization with cardiopulmonary bypass (CPB) and the use of double internal mammary artery grafts^3^.Thus, over the course of 40 years, CABG has undergone considerable development favoring this surgical practice. However, over the last 20 years there has been a significant change in the profile of patients undergoing CABG, which is usually performed with older individuals with more severe heart damage, most often with comorbidities associated with Cardiovascular Disease (CVD), representing greater risk for reoperations, complications and mortality^4^. 

The mortality rate is usually applied by health services as a quality indicator, however, this indicator is often used in a generalized way, without knowing the exact profile of the population studied. In an attempt to identify mortality risks related to CABG, seeking to understand the factors that influence the outcome of this surgical practice is essential for dealing with CVD. Although extensive literature shows data on the mortality rates of patients undergoing CABG^5^ ^-^ ^6^, few studies portray the factors associated with mortality in this type of surgery. 

In the health area, nursing is the only profession that includes, in its formation, theory combined with practice in relation to health management or administration^7^. In this sense, the use of reliable indicators is essential for the effective and efficient management of care, since this allows the identification of avoidable risks, subsidizes corrective action planning and directs prevention strategies for events and complications related to CVD. The potential of the professional nurse to contribute to the changing reality of chronic diseases should be noted, particularly regarding CVD, by identifying risk factors and preventing potential events that complicate the state of health of the individual affected by CVD, with the aim of ensuring the quality of the care provided^8^. 

Given the previously mentioned considerations, the following question arises: What factors are associated with the occurrence of mortality in patients undergoing coronary artery bypass grafting in a cardiology referral institution of Santa Catarina? In relation to the above question, the aim of this study was to investigate the factors associated with mortality in patients undergoing coronary artery bypass grafting in a cardiovascular referral hospital of Santa Catarina. 

## Method

The study had a quantitative, exploratory, descriptive and retrospective focus and was performed in a public, cardiovascular referral hospital of Santa Catarina. Data were gathered from the documents, records and statistical data of the institution related to the patients who underwent CABG in the previous nine years, i.e., between 2005 and 2013.

Data collection was carried out in three stages. Firstly, the record of CABGs conducted in the institution within the study period was obtained from the surgical center. Next, the list of deaths that occurred during the same period was requested from the statistical sector of the institution. The manual organization of the data resulted in a single table in Microsoft Excel^(r)^ with the record of the patients that underwent CABG and those who died after performing the surgery. In the third stage, data collection was carried out in the medical records of those patients identified in the previous step, totaling 1447 patient records. No medical record was excluded considering that the data collection period was established due to the quality in completing the information of the forms. At this time, the factors associated with mortality were sought, which in this study refer to the profile of patients who underwent CABG, in the period between 2005 and 2013, including: gender, age, skin color, cardiopulmonary bypass and type of surgery (CABG alone or combined with other surgery), and specific data of patients who died due to CABG, such as: hospitalization diagnosis, risk factors for coronary artery disease (CAD), complications recorded during hospitalization, length of hospitalization and the main cause of death. Thus, the variables of the groups: profile, main diagnosis, risk factors, complications recorded during hospitalization and length of hospitalization were crossed with the main cause of death group.

Data were analyzed using descriptive (means, medians, standard deviation, minimum and maximum amplitude) and inferential statistics, using the Fisher-Freeman-Halton Exact Test to compare the percentage between groups; Student's t-test to compare continuous variables; and analysis of variance followed by Tukey's comparisons to compare the mean of more than two groups. In addition, the odds ratio was also calculated. For the statistical tests, a significance level of 0.05 was assumed, equivalent to a 95% confidence interval. For this analysis the SAS version 9 software was used, with help from a statistician. 

This study was related to the project entitled "The revascularized cardiac patient: the process of referral and counter referral of the health services of Santa Catarina" approved by the Human Research Ethics Committee of the Federal University of Santa Catarina (CEPSH/USFC) under number 120.184 in 2012. Thus, for the performance of this study an amendment to the main project was made, aiming to obtain approval from CEPSH/UFSC to collect data in the medical records. The study followed Resolution No. 466/2012 of the National Health Council/Ministry of Health, which provides Guidelines and Regulatory Norms for Research Involving Human Subjects.

## Results

In the period from 2005 to 2013, 1447 patients underwent CABG in the institution chosen for this study. 

A total of 455 patients (31.4%) were female and 992 (68.6%) male. Regarding skin color, 1104 (76.45) patients were declared white and 26 (1.7%) black, with no information for 317 (21.9%). Of all the patients, 797 (55%) underwent CABG with CPB and 650 (45%) underwent surgery without CPB. Regarding the type of surgery, it was found that the majority of the patients, 1302 (89.9%), underwent CABG without any other associated surgery. Only 145 (10.1%) patients underwent CABG associated with another cardiac surgery. The results show a certain balance between the number of people that underwent CPB or not (Table 1).

**Table 1:** Distribution of patients according to gender, skin color, CPB and type of surgery in relation to death. São José, SC, Brazil, 2005-2013.

Characterization of the patients.		Death - n (%)	P-value	Odds Ratio	CI OR*
Gender					
	Female	31(6.8%)	0.076	1.54	(0.96 ; 2.47)
	Male	45(4.5%)			
Skin color					
	Black	3 (11.5%)	0.184	2.19	(0.64 ; 7.50)
	White	62 (5.6%)			
	No information	11 (3.4%)			
CPB^†^					
	Present	62 (7.8%)	<0.001^§^	3.84	(2.13 ; 6.92)
	Absent	14 (2.2%)			
Type surgery					
	CABG^‡^ only	54 (4.2%)	<0.001^§^	4.12	(2.43 ; 7.00)
	CABG associated with other surgeries	22 (15.1%)			

* Confidence Interval for Odds Ratio † Cardiopulmonary Bypass; ‡ Coronary Artery Bypass Grafting; § Statistically Significant Difference (p<0.05).

With regard to mortality, of the total number of patients who underwent surgery during the study period (1447), 1371 (94.7%) survived and 76 (5.3%) died. Thus, the analysis of the relationship between the number of deaths and variables in this study was carried out. 

When the annual mortality rates were considered, there was some increase over the years, especially when comparing the year 2005, in which the mortality rate was 1.1%, and the year of 2012, in which this was 13.1%, representing an increase of 12%. In 2013 there was a reduction in mortality from 13.1% to 10.5%.

Regarding the age of the individuals undergoing CABG, the mean was 60.6 years, median 61 years, standard deviation 9.6 years, minimum 18 years and maximum 86 years. The results showed a great variability, with ages between 30-85 years. For those who died, the mean age was 65.1 years, median 67.0 years, standard deviation 10.5 years, minimum 38 years and maximum 86 years. 

To verify whether there was a significant difference between the mean ages, Student's t-test for independent samples was performed and the descriptive level <0.001 obtained. From this, it was concluded that the mean of group of deaths was significantly higher than the group without death. The Odds Ratio for age was 1.0566 for each year. Considering 10 years, the Odds Ratio was 1.73, that is, the chance of death with 10 more years of age is 1.73 times higher.

Considering associated surgeries, there was some difference in the percentage of deaths with regard to CABG associated with mitral valve replacement (MVR), presenting a higher percentage (26.7%), followed by CABG associated with aortic valve replacement (AVR) (16.0%). Calculating the Odds Ratio for each associated surgery, compared to CABG only, it was found that for CABG associated with AVR the Odds Ratio was 4.39 and for CABG associated with MVR the Odds Ratio was 8.38, showing an increased chance of death for CABG associated with MVR. Thus, it can be concluded that there was a significant difference in the mortality according to the type of surgery.

In relation to the main cause of death for the study population, 29 (38.2%) patients died due to cardiogenic shock, 16 (21.1%) from multiple organ failure, 5 (6.6%) acute myocardial infarction (AMI), 5 (6.6%) septic shock, and 4 (5.3%) due to cardiac arrest (CA). The other causes combined accounted for 17 (22.4%) patients.

The majority of the patients hospitalized for CABG had AMI as the main diagnosis, 36 (47.3%) patients, followed by unstable angina (UA), 17 (22.3%) patients, and congestive heart failure (CHF) and heart failure (HF), both with 9 (11.8%) patients. The other diagnoses combined accounted for 5 (6.5%) patients. 

Regarding the distribution of the causes of death by main hospitalization diagnoses, there was a tendency for the diagnosis of CHF, 5 (55.6%) patients, UA, 8 (47.1%) patients, and AMI, 13 (36.1%) patients. The percentage of death from cardiogenic shock was not significant, as shown in Table 2. To verify whether the percentages of causes of death were the same for the diagnostic groups, Fisher's exact test was carried out and the descriptive level of 0.691 obtained, which showed no significant differences between the diagnostic groups.

**Table 2:** Distribution of the main cause of death and hospitalization diagnosis. São José, SC, Brazil, 2005-2013.

Hospitalization diagnosis		Cardiogenic shock	Septic shock	Multiple Organ Failure	Acute myocardial infarction	CA*	Others
UA^†^	N	8	1	3	1	1	3
	%	47.1	5.9	17.6	5.9	5.9	17.6
	CI^‡^	(26.2;69.0)	(1.0;27.0)	(6.2;41.0)	(1.0;27.0)	(1.0;27.0)	(6.2;41.0)
AMI^§^	N	13	1	8	4	3	7
	%	36.1	2.8	22.2	11.1	8.3	19.4
	CI	(22.5;52.4)	(0.5;14.2)	(11.7;38.1)	(4.4;25.3)	(2.9;21.8)	(9.8;35.0)
CHF‡	N	5	1	0	0	0	3
	%	55.6	11.1	0.0	0.0	0.0	33.3
	CI	(26.7;81.1)	(2.0;43.5)	(0.0;29.9)	(0.0;29.9)	(0.0;29.9)	(12.1;64.6)
HF^¶^	N	2	2	2	0	0	3
	%	22.2	22.2	22.2	0.0	0.0	33.3
	CI	(6.3;54.7)	(6.3;54.7)	(6.3;54.7)	(0.0;29.9)	(0.0;29.9)	(12.1;64.6)
Others	N	1	0	3	0	0	1
	%	20.0	0.0	60.0	0.0	0.0	20.0
	CI	(3.6;62.4)	(0.0;43.4)	(23.1;88.2)	(0.0;43.4)	(0.0;43.4)	(3.6;62.4)
Total	N	29	5	16	5	4	17
	%	38.2	6.6	21.1	6.6	5.3	22.4
	CI	(28.1;49.4)	(2.8;14.5)	(13.4;31.5)	(2.8;14.5)	(2.1;12.8)	(14.5;32.9)

*Cardiac Arrest; † Unstable Angina; ‡ Confidence Interval; § Acute Myocardial Infarction; || Congestive Heart Failure; ¶ Heart Failure.

The main risk factors associated with CAD presented by the study population were hypertension (HT), 59 (78.7%) patients, smoking, 39 (52%) patients, dyslipidemia, 31 (41.3% ) patients, and diabetes mellitus (DM), 30 (40%) patients. For the distribution of causes of death (according to each risk factor) Fisher's exact test was applied and a trend between the HT risk factor and death due to CA (100%) was seen, followed by death due to multiple organ failure (87.5%) and cardiogenic shock (72.4%), however, this was not significant. Based on Table 3, it was concluded that the smoking and dyslipidemia risk factors were closely related to death from AMI (75%) and CA (75%). The descriptive level showed no significant difference between the diagnostic groups.

**Table 3:** Distribution of causes of death according to each CAD risk factor. São José, SC, Brazil, 2005-2013.

Cause of death	Smoking			Hypertension			Diabetes			Dyslipidemia		
	No	Yes	CI^*^	No	Yes	CI	No	Yes	CI	No	Yes	CI
Cardiog shock^†^	14	15	(34.4;68.6)	8	21	(54.3;85.3)	18	11	(22.7;56.0)	20	9	(17.3;49.2)
Septic shock	3	2	(11.8;76.9)	2	3	(23.1;88.2)	3	2	(11.8;76.9)	4	1	(3.6;62.4)
MOF^‡^	11	5	(14.2;55.6)	2	14	(64.0;96.5)	8	8	(28.0;72.0)	9	7	(23.1;66.8)
AMI^§^	1	3	(30.1;95.4)	2	2	(15.0;85.0)	3	1	(4.6;69.9)	1	3	(30.1;95.4)
CA^||^	1	3	(30.1;95.4)	0	4	(51.0;100.0)	2	2	(15.0;85.0)	1	3	(30.1;95.4)
Others	6	11	(41.3;82.7)	2	15	(65.7;96.7)	11	6	(17.3;58.7)	9	8	(26.2;69.0)
Total	36	39	(40.9;62.9)	16	59	(68.1;86.4)	45	30	(29.7;51.3)	44	31	(30.9;52.6)
p-value			0.3491			0.2511			0.9381			0.3102

*Confidence Interval; †Cardiogenic Shock; ‡Multiple Organ Failure; §Acute Myocardial Infarction; ||Cardiac Arrest.

Table 4 presents the complications recorded during the hospitalization, grouped into four areas: cardiovascular system, represented by 53 patients, respiratory system, 34 patients, renal system, 31 patients and infections, 27 patients. Fisher's exact test was performed and in general the descriptive level of 0.1471 was obtained, which showed a tendency between the cardiovascular system variable and death, however, the difference was not significant.

**Table 4:** Distribution of causes of death for each general complication group. São José, SC, Brazil, 2005-2013.

Cause of death	Cardiovascular System			Respiratory System			Renal System			Infections		
	No	Yes	CI*	No	Yes	CI	No	Yes	CI	No	Yes	CI
Cardiog shock^†^	10	19	(47.3;80.1)	18	11	(22.7;56.0)	20	9	(17.3;49.2)	25	4	(5.5;30.6)
Septic shock	1	4	(37.6;96.4)	1	4	(37.6;96.4)	2	3	(23.1;88.2)	0	5	(56.6;100.0)
MOF^‡^	1	15	(71.7;98.9)	5	11	(44.4;85.8)	5	11	(44.4;85.8)	7	9	(33.2;76.9)
AMI§	2	3	(23.1;88.2)	5	0	(0.0;43.4)	5	0	(0.0;43.4)	5	0	(0.0;43.4)
CA||	2	2	(15.0;85.0)	4	0	(0.0;49.0)	3	1	(4.6;69.9)	3	1	(4.6;69.9)
Others	7	10	(36.0;78.4)	9	8	(26.2;69.0)	10	7	(21.6;64.0)	9	8	(26.2;69.0)
Total	23	53	(58.7;78.9)	42	34	(34.1;55.9)	45	31	(30.4;52.0)	49	27	(25.7;46.7)
p-value			0.1471			0.0118			0.0459			0.0002

*Confidence Interval; †Cardiogenic Shock; ‡Multiple Organ Failure; §Acute Myocardial Infarction; ||Cardiac Arrest.

The complications of the cardiovascular system showed a tendency with all the causes of death. Among them, arrhythmia (75%) and AMI (50%) showed a certain tendency with regard to death due to AMI. Regarding the distribution of causes of deaths for respiratory complications, pleural effusion (60%) and pneumonia/bronchopneumonia (60%) showed a tendency with death due to septic shock. Regarding the distribution of causes of death for each complication of the renal system, acute renal failure (ARF) (62.5%) presented a tendency with death due to multiple organ failure. In relation to the distribution of causes of death by infections, only death from septic shock showed a tendency. 

**Figure 1:**
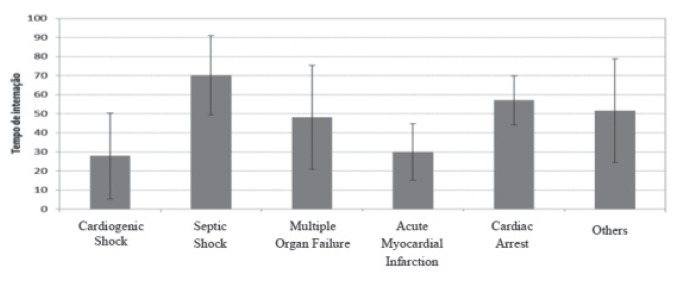
Mean ± 1 standard deviation for time of hospitalization and the main cause of death

The length of hospitalization is presented in Figure 1, which ranged from 1 to 98 days, with means of 27.9 and 70.2 days depending on the cause of death. 

Regarding the length of hospitalization and cause of death, higher means of hospitalization can be seen to be related to death from septic shock, mean of 70.2 days, median 72 days, standard deviation 20.8 days, minimum of 39 days and maximum of 92 days.

## Discussion

The results demonstrate that the mortality rate of the study institution (5.3%) was slightly above the national mean of 4.8%^3^. With regard to the profile of patients that undergo CABG, in a study conducted in southern Brazil, the age varied from 32 to 86 years and the age group with the highest incidence was between 51 and 70 years, in which 688 individuals (68.5%) were male^9^. This information confirms the findings of the present study and the tendency for indication for CABG of an older, male population.

The high mortality rate was influenced by the final two years of the study, 2012 and 2013, in which mortality rates were 13.1 and 10.5%. This may be related to the hiring of new professionals in the institution with less experience, causing change in the medical teams and nursing practitioners, as well as the indication for CABG of more clinically severe and older subjects, highlighting the need for CVD prevention strategies.

Despite the predominant population for CABG indication being male, mortality compared by gender, in this study, showed a higher rate of death for women. A study of cardiac surgeries performed in the SUS nationwide, showed a lower mortality rate for males compared to females, 5.20% versus 8.25% (p<0.001)^1^. Therefore, it can be stated that being female is a clinical-demographic characteristic associated with mortality in CABG^10^.

Older age appears as a risk factor for mortality in patients undergoing CABG. A study conducted in Brazil with, mostly male (56.3%), octogenarian patients that all underwent CABG with CPB, showed a mortality rate within the hospital of 14.8%, more than double the national mean. It cited cardiogenic shock (42.8%) as the main cause of death^11^, in agreement with the findings of this study, which showed cardiogenic shock as the main cause of death, with an increased risk of death of 1.73 for every 10 more years of age. 

Regarding race, studies in southern Brazil present a predominance of white patients with CABG indication, 68.9%^10^ ^)^ and 95.7%^12^ ^)^ in the states of Santa Catarina and Rio Grande do Sul, respectively. However, it is pertinent to consider that these studies were conducted in southern Brazil, where the population is predominantly Caucasian. In a study conducted in northeastern Brazil, which sought to identify the sociodemographic characteristics of patients undergoing CABG, 53% of the patients were declared non-white^13^. In relation to the higher mortality rate in black patients undergoing CABG, there is evidence of a higher rate of deaths from circulatory system diseases in black men, as well as the association of death with the prevalence of hypertension in this race^14^.

The results of this study also indicate a higher chance of death for CABG associated with other surgeries, especially valve replacement surgery. A study conducted in southern Brazil that investigated preoperative risk factors for heart valve surgery found a significant increase in the rate of death, from 8.8% to 25%, in patients undergoing this procedure^15^.

The use of CPB in CABG demonstrated a significantly higher chance of mortality compared to its non-use. Conventional CABG in CPB is associated with a significant risk of related morbidity and mortality in older patients^16^. Other studies show the use and duration of CPB in CABG as a factor related to mortality within the hospital^10^. 

Another study confirmed the results presented here with regard to hypertension as the main risk factor for CHD related to death in patients undergoing CABG. In this same study, half of the patients were affected by ACS, in which UA appeared more often in relation to AMI, differing from the findings discussed that identified AMI as the main hospitalization diagnosis^17^. Dyslipidemia, smoking, physical inactivity, obesity, diabetes mellitus (DM) and unhealthy diets are cited as potential aggravating factors for impairment of health and installation of complications in CAD^1^. 

It should be highlighted that complications related to the cardiovascular system were more representative, in agreement with a study developed in southeastern Brazil, in which 24.5% of the patients presented cardiovascular changes. Pulmonary infection was the most frequent infectious complication (15.3%), corroborating the findings of this study that showed the occurrence of pneumonia or bronchopneumonia in patients who had death related to infection. The infected patients had longer periods of hospitalization in relation to those who did not have this occurrence recorded during the hospitalization^18^.

It was found that the mean length of hospitalization presented a consistently heterogeneous variable, with a minimum of 1 day and up to 45 days, with the hospitalization period exceeded 11 days in 25% of the cases, which indicates treatment of complex events with long stays and higher hospital costs^19^. In another study, the vast majority of patients were operated electively, after waiting a long time for the surgery, with a mean of 35.5 days^5^. Both confirm the findings of this study with regard to the prolonged period of hospitalization. Thus, it is relevant for the mortality indicator to be used by health professionals and managers. Considering that, among the various professions in the health area, nurses stand out for having specific courses in their formation aimed at health management and planning, they can potentiate the use of these indicators in their practice to improve the quality of the care provided, abandoning unplanned actions and realizing the need to have a plan^20^.

Despite the development of interventionist actions for the individual with CVD, in high-complexity health, nursing care should mainly be based on prevention. The administration of nursing care brings the individual close to the professional, as it considers the health-disease process in a broad way. Thus, the search for new perspectives and strategies reaffirms the importance of nursing in the care for individuals affected by CVD and sometimes submitted to CABG, as the aim is to promote well-being and quality of health of these patients, through the awareness of the existence of their problems and their consequent complications^21^. 

## Conclusion

In this study, the main factors associated with the occurrence of death in patients undergoing CABG were: people with advanced age; use of CPB; occurrence of associated CABG; manifestation of infection and prolonged hospitalization period. Considering that these factors were shown to directly impact on the mortality of patients, greater attention of health staff and managers to this profile is indicated. The results of this study provide subsidies for the nursing practice with preventive measures for the CVD event, as well as in prevention and early identification of complications associated with CABG. 

As limitations, the study did not address aspects frequently discussed in the literature that could influence the mortality of patients undergoing CABG, such as length of surgery, length of CPB and intraoperative complications. The reason for this was that the study proposal was to work with aspects still little discussed in the literature, in order to seek other associations with the occurrence of death after CABG.
